# Gastric Volvulus: A Rare Cause of Intestinal Occlusion

**DOI:** 10.7759/cureus.57591

**Published:** 2024-04-04

**Authors:** Majdoubi Amine, El Hammouti Mohammed, El Achchi Anass, Tariq Bouhout, Badr Serji

**Affiliations:** 1 Surgical Oncology Department, Regional Oncology Center, Mohammed VI University Hospital, Faculty of Medicine and Pharmacy, Mohammed First University, Oujda, MAR

**Keywords:** bowel obstruction, fundoplication, gastropexy, organoaxial volvulus, gastric volvulus

## Abstract

Gastric volvulus (GV) is a rare condition characterized by the rotation of all or part of the stomach around its transversal or longitudinal axis. We report the case of a 76-year-old woman with the acute form of GV, likely exacerbated by hiatal hernia and age-related ligamentous relaxation, evolving for a week before her admission. She underwent a midline laparotomy with fundoplication at 270° and anterior gastropexy. GV poses life-threatening risks in its acute presentation. Surgical management entails restoring the hemodynamic stability, surgical detorsion of the volvulus, and addressing the etiology (hiatal hernia).

## Introduction

Gastric volvulus (GV) is an abnormal rotation of all or part of the stomach around its longitudinal or transverse axes, leading to gastric dilation with occlusion, strangulation, ischemia, and gastric necrosis [1]. Depending on its axis of rotation, GV is classified into organo-axial (along the longitudinal axis), mesentero-axial (along the transverse axis), and mixed volvulus [2]. It is a rare and poorly understood pathology exacerbated by hiatal hernia and ligament laxity. Its management requires collaboration between intensivists, surgeons, and gastroenterologists. It manifests in the following three clinical forms of increasing severity: chronic, intermittent, and acute. The acute form is a life-threatening condition and poses a management challenge. Treatment aims to stabilize the hemodynamic parameters, followed by surgical reduction of the volvulus and its etiology (prevention of recurrences) [3].

Here, we report the case of a 76-year-old woman who presented to the emergency room with an acute form of GV. In addition, we discuss the clinical, radiological, and therapeutic aspects of this rare pathological condition.

## Case presentation

A 76-year-old woman with a history of symptomatic hiatal hernia, pericarditis, and well-controlled diuretic right heart failure regularly took antacids and proton pump inhibitors to relieve symptoms of gastroesophageal reflux. There was no smoking, alcoholic consumption, or surgical history, and there was no history of cancer in the family. The chronology of the disease went back six months before her admission, with the start of epigastric pain associated with episodes of vomiting, for which the patient consulted a gastroenterologist and benefited from gastroscopy with biopsies. The biopsy showed a hiatal hernia with *Helicobacter pylori* gastritis treated with the sequential treatment of *H. pylori*, without confirming its elimination. The evolution of the symptoms was characterized by the recurrence of intermittent painful episodes in the epigastric region. Up to a week before her admission, the patient experienced acute atypical epigastric pain with nausea, vomiting, weight loss, and deterioration of the general condition. However, the patient denied having gas, stool, fever, or intestinal bleeding in the last 24 hours of fever.

At the emergency department, the patient was stable with the following hemodynamic parameters: Glasgow Coma Scale score of 15/15, blood pressure of 120/95 mmHg, pulse of 90 beats/minute, temperature of 37.1°C, respiratory rate of 18 cycles/minute, and SpO_2_ of 100% in ambient air.

Physical examination revealed distension and lapping in the upper abdomen, with no tenderness on palpation, and the epigastric region to the peri-umbilical regions was tympanic to the percussion of the abdomen. Intestinal noises at abdominal auscultation were exaggerated with no signs of peritonitis, and the insertion of the nasogastric tube was impossible due to the pain and regurgitation.

After this assessment, the initial diagnosis was high intestinal obstruction based on symptoms and clinical signs without biological repercussions (Table 1).

**Table 1:** Blood test results.

Biological parameters	Value	Normal range
Leukocyte (mm^3^)	12,000	4,000–10,000
Hemoglobin (g/dL)	10.5	135–145
Potassium (mmol/L)	4.0	3.5–5.0
Sodium (mmol/L)	140	135–145
C-reactive-protein (mg/L)	20	<4
Aspartate transferase (IU/L)	17	5–34
Gamma-glutamyltransferase (IU/L)	12	9–36
Alkanine phosphatase (IU/L)	60	40–150
Total bilirubin (mg/L)	7	2–12
Direct bilirubin (mg/L)	4	0–5
Urea (mmol/L)	0.24	0.15–0.45
Creatinine (μmol/L)	6.86	5.7–11.1
Prothrombin (%)	96	70–100
Lipase (IU/L)	103	<78
Lactate ( mmol/L)	1.23	0.5–1.5
pH	7.37	7.35–7.45
pCO_2_ (mmHg)	42	35–45
HCO_3¯_ (mmol/L)	23	22–28

**Figure 1:**
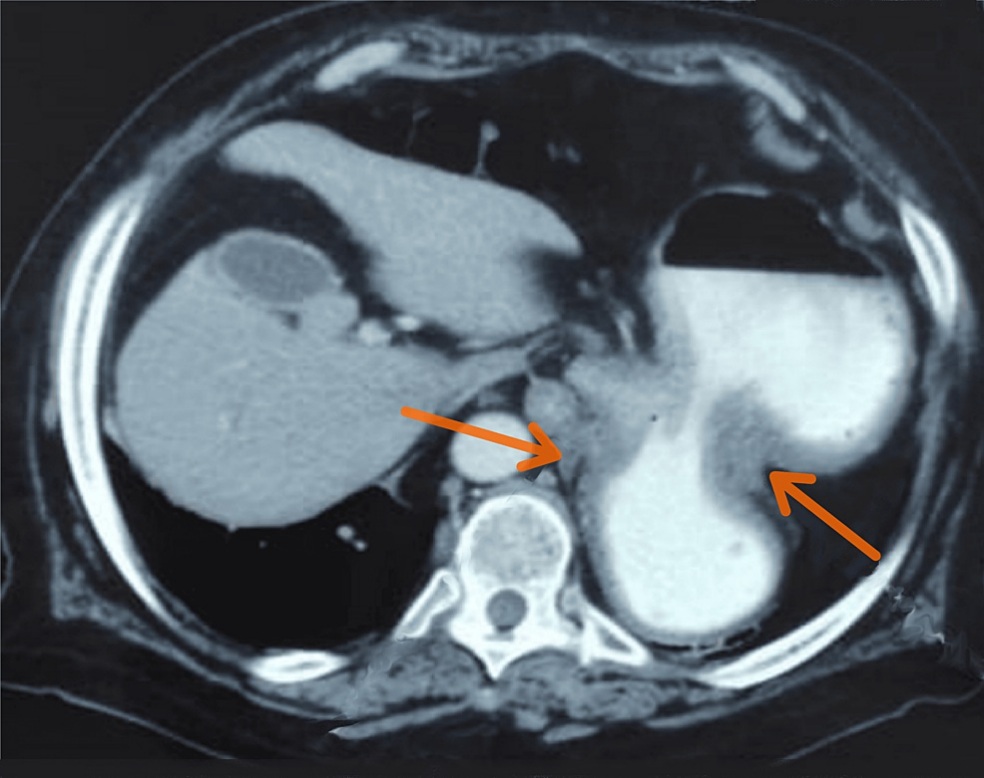
Axial section of the abdominal CT showing a distended stomach with torsion of the gastric body (two arrows) and upper air-fluid level.

The patient underwent an abdominopelvic computed tomography (CT), which revealed an uneven expansion of the stomach with a twist of the gastric body along its longitudinal axis, resulting in a delay of gastric emptying, with an air-fluid level (Figure 1), which suggested an organo-axial GV.

In a few hours, the patient presented sudden-onset hemodynamic instability with a drop in blood pressure to 90/60 mmHg and tachycardia at 108 beats/minute. After rapid medical resuscitation, she underwent an exploratory midline laparotomy by our professor with 15 years of experience in a university hospital under general anesthesia, and the nasogastric tube evacuated about 1 L of gastric content.

**Figure 2:**
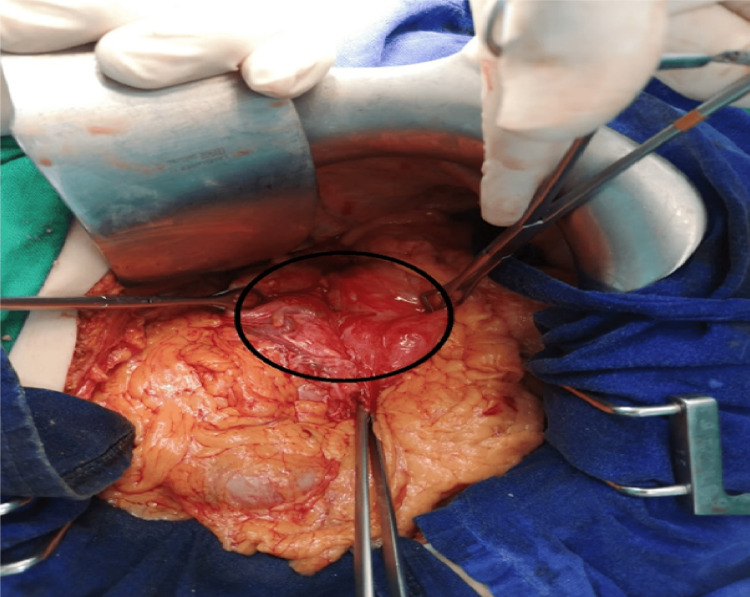
Operative image showing the strangled stomach (black circle).

The operative explorations showed an organo-axial rotation of the stomach with multiple adhesions without signs of gastric necrosis (Figure 2). After a tedious and gradual dissection of the stomach, inflammatory tissue, and adhesions around the volvulus site, the stomach was rotated counterclockwise along its axis to release the twist until it regained its anatomical position. A 270° fundoplication was performed by rotating the fundus backward and attaching it to the middle part of the esophagus and anterior gastropexy by anchoring the anterior wall of the stomach to the abdominal wall with a resorbable suture.

The postoperative period was uneventful, and the patient was released after seven days of hospitalization. Follow-up for two and a half years showed good evolution without complications or recurrence.

## Discussion

GV is a rare form of bowel occlusion, and Ambroise was the first to describe it in 1579 by reporting the result of an autopsy in a person with a sword wound, a case of strangulated diaphragmatic hernia with intrathoracic GV [3]. GV refers to the rotation of all or part of the stomach relative to one of its longitudinal or transversal axes, thus creating the conditions for gastric dilation with high occlusion, strangulation, ischemia, and gastric necrosis [1]. The age range most affected is in the fifth decade of life, and about 10-20% of cases occur in the pediatric population [4].

Risk factors for GV in adults include age over 50 years, diaphragmatic abnormalities such as hiatal hernias, Larrey’s slit hernia, Bochdalek’s hernias, diaphragmatic eventration, traumatic diaphragmatic hernia, phrenic nerve paralysis, as well as other gastrointestinal or splenic anatomical abnormalities such as “wandering spleen” or “bell clapper” spleen and kyphoscoliosis [5].

The symptoms of GV depend on the degree of strangulation and the type of volvulus. Consequently, three clinical forms have been described. The acute form, the most severe form, is associated with stomach strangulation, manifested by the Borchardt triad that occurs in about 70% of cases, comprising acute pain, vomiting, and inability to introduce a nasogastric tube [6]. Hematemesis may also be present due to mucosal bleeding from ischemia or mucosal tears due to vomiting [7]. In the chronic form, the most frequent symptomatology is non-specific. The stomach may volvulate and remain partially obstructed, resulting in incomplete and chronic obstruction [7]. The intermittent form is dominated by recurrent episodes of acute obstruction that resolve spontaneously and abruptly as the stomach returns to its normal position [7].

CT is the recommended diagnostic modality, providing comprehensive assessment and characterization of GV, encompassing type, etiology, and associated cardiopulmonary consequences arising from intrathoracic herniation. Using sagittal reconstructions facilitates precise identification of rotational direction, torsion point, and anatomical factors contributing to volvulus, while also detecting potential signs of parietal necrosis, thus guiding timely surgical intervention [7].

Millet et al. [7] suggested that the most sensitive direct signs of gastric volvulus were an antropyloric transition point without any abnormality at the transition zone and the antrum at the same level or higher than the fundus (the inversed position of large and small gastric curvatures and stenosis of gastric segments through the stretched esophageal hiatus). The presence of these two findings as diagnostic criteria of GV had 100% sensitivity and specificity for the diagnosis of GV.

Gastroscopy’s diagnostic efficacy in detecting GV is limited; however, specific indicators may suggest its presence. Distortions in gastric anatomy, coupled with the difficulty or inability to visualize the pylorus, may indicate GV [8].

The management of GV involves two parts. First, it entails optimization through medical preparation, consisting of hydroelectrolytic resuscitation and the placement of a nasogastric tube for early stomach decompression [9]. The administration of broad-spectrum intravenous antibiotics is recommended because of the potential risk of gastric necrosis and mediastinitis or as part of prevention from infection at the operative site [5]. The second consists of surgical treatment; acute GV remains an emergency surgical repair. In patients who are not candidates for surgery (due to comorbidities or inability to tolerate anesthesia), endoscopic reduction may be attempted in the absence of perforation and necrosis [9]. Chronic gastric volvulus is treated without urgency.

The basis for surgical treatment of GV includes decompression, reduction, and prevention of recurrence. Tanner described several surgical options for GV repair [10], which include diaphragmatic hernia repair, simple gastropexy, gastropexy with gastrocolic epiploon division (Tanner’s operation), partial gastrectomy, fundoantral gastrostomy (Opolzer’s operation), and repair of a diaphragm eventration.

Laparoscopic surgery is frequently indicated, particularly in acute cases, as it provides access to the abdominal cavity [11,12]. It offers the advantage of facilitating additional procedures such as organ reintegration into the intra-abdominal space, partial or total gastrectomy, gastrostomy, and diaphragmatic exploration. In open surgery, the conventional surgical approach for GV involves performing an emergency laparotomy with anterior gastropexy. Nevertheless, partial or total gastrectomy may be required in necrosis or gastric perforation [11].

Although there is a lack of data from randomized trials comparing open surgery and laparoscopic surgery in GV, several studies have shown results in favor of the laparoscopic approach over open surgery [13].

## Conclusions

GV is a rare and poorly understood condition. Ligament laxity and diaphragmatic hernias are consistent etiological factors, irrespective of the type and form of volvulus. Surgical management adheres to fundamental principles, encompassing decompression, reduction, and prevention of recurrence of GV.

In emergencies, laparoscopic intervention frequently demonstrates enhanced outcomes relative to laparotomy owing to its improved access and facilitation of supplementary procedures within the abdominal cavity. For patients deemed ineligible for surgical intervention due to comorbidities or anesthesia intolerance, endoscopic reduction may present a viable alternative. This strategy warrants assessment before embarking on definitive repair of the volvulus and its underlying etiology, contingent upon reevaluation of the patient’s clinical condition.
